# Identification of Predictor Genes for Feed Efficiency in Beef Cattle by Applying Machine Learning Methods to Multi-Tissue Transcriptome Data

**DOI:** 10.3389/fgene.2021.619857

**Published:** 2021-02-16

**Authors:** Weihao Chen, Pâmela A. Alexandre, Gabriela Ribeiro, Heidge Fukumasu, Wei Sun, Antonio Reverter, Yutao Li

**Affiliations:** ^1^College of Animal Science and Technology, Yangzhou University, Yangzhou, China; ^2^CSIRO Agriculture and Food, St Lucia, QLD, Australia; ^3^School of Animal Science and Food Engineering, University of São Paulo, Pirassununga, Brazil; ^4^Institute of Agriculture Science and Technology Development, Yangzhou University, Yangzhou, China; ^5^Joint International Research Laboratory of Agriculture and Agri-Product Safety of Ministry of Education of China, Yangzhou University, Yangzhou, China

**Keywords:** residual feed intake, *Bos indicus*, co-expression network, RNA-seq, Random Forest, Extreme Gradient Boosting, supporting vector machine

## Abstract

Machine learning (ML) methods have shown promising results in identifying genes when applied to large transcriptome datasets. However, no attempt has been made to compare the performance of combining different ML methods together in the prediction of high feed efficiency (HFE) and low feed efficiency (LFE) animals. In this study, using RNA sequencing data of five tissues (adrenal gland, hypothalamus, liver, skeletal muscle, and pituitary) from nine HFE and nine LFE Nellore bulls, we evaluated the prediction accuracies of five analytical methods in classifying FE animals. These included two conventional methods for differential gene expression (DGE) analysis (*t*-test and edgeR) as benchmarks, and three ML methods: Random Forests (RFs), Extreme Gradient Boosting (XGBoost), and combination of both RF and XGBoost (RX). Utility of a subset of candidate genes selected from each method for classification of FE animals was assessed by support vector machine (SVM). Among all methods, the smallest subsets of genes (117) identified by RX outperformed those chosen by *t*-test, edgeR, RF, or XGBoost in classification accuracy of animals. Gene co-expression network analysis confirmed the interactivity existing among these genes and their relevance within the network related to their prediction ranking based on ML. The results demonstrate a great potential for applying a combination of ML methods to large transcriptome datasets to identify biologically important genes for accurately classifying FE animals.

## Introduction

As farm practices around the world are continuously challenged to minimize environmental footprint, there is a growing need for livestock producers to identify and select superior animals for efficiency-related traits (Hayes et al., 2013). Among those, feed efficiency (FE) is one of the traits that can be used to increase productivity while decreasing both pollutant production and competition for high-quality grains with human nutrition (Banerjee et al., 2020; Yang et al., 2020). However, FE is a complex trait, not only regulated by several biological processes, but also presented a moderate heritability in beef cattle (Archer et al., 1997; Arthur et al., 2018; Higgins et al., 2019), suggesting a great influence of environmental effects (e.g., diet and management). Considering that diverse mechanisms are involved in FE regulation, it is often difficult to develop molecular markers that accurately differentiate animals between high FE (HFE) and low FE (LFE), when using a traditional case-control study method. That is because, unlike healthy vs diseased or treated vs non-treated contrasts, differences between HFE and LFE are subtle and often related to intrinsic metabolic processes (Cantalapiedra-Hijar et al., 2018). For instance, animals from both groups can be healthy, of the same age, same breed, under the same management and nutritional conditions, only differing in the amount of food they consume *ad libitum* (Alexandre et al., 2015; Russell et al., 2016). Therefore, the development and application of accurate methods to identify predictor molecules of polygenic traits, such as FE, are essential for the implementation of an effective genomic selection program in livestock species.

Decreasing costs and increased accessibility to high-throughput sequencing technologies have enabled the generation of larger RNA sequencing (RNA-seq) datasets aiming to investigate predictor genes of complex traits, such as FE. In this context, machine learning (ML) has been shown to outperform other approaches when analyzing large RNA-seq datasets, and selecting subsets of candidate genes for the prediction or classification of phenotypes (Thompson et al., 2016; Choi et al., 2018; Wang et al., 2018). To date, several studies have reported the application of different ML methods in prediction for FE. For example, Messad et al. (2019) successfully tested the reliability of gradient tree boosting (XGBoost) in identifying molecular predictors of FE in pigs; Yao et al. (2013) found that Random Forest (RF) could be used effectively to identify additive predictors associated with FE in cattle; and support vector machine (SVM) had also been proven to be a reliable method in genomic prediction of FE in dairy cattle (Yao et al., 2016). Piles et al. (2019) found that out of four ML methods used [RF, SVM, Elastic Net (ENET), and nearest shrunken centroid], ENET produced the best classification accuracy of residual feed intake (RFI) in pigs using 200 selected genes from liver. However, in all these studies, ML methods were evaluated individually, and none has focused on the comparison of the performance of combining different ML methods together in the prediction of HFE and LFE animals. In other words, the full advantage of joint forces of different ML methods has not been thoroughly investigated.

In this study, using RNA-seq data of five tissues from nine HFE and nine LFE Nellore bulls, we aimed to evaluate the prediction accuracies of six analytical methods in classifying animals as either HFE or LFE. For comparison purposes, these included four ML methods [RF, XGBoost, combination of both RF and XGBoost (RX), and SVM] and two conventional methods for differentially expressed genes (DEG) identification (*t*-test and edgeR). Furthermore, co-expression network and functional enrichment analyses were conducted to ascertain the biological relevance of the potential predictor genes identified from the methods with strongest prediction accuracy. This study enhances our current knowledge about the performance of different ML methods in identifying predictor genes for assigning animals to phenotype groups. Most importantly, it demonstrates that a combination of ML methods is the best approach to investigate traits of economic and environmental relevance.

## Materials and Methods

### Transcriptome Dataset

The transcriptome dataset used in this study is publicly available in the European Nucleotide Archive under the study ID PRJEB27337.^1^ Detailed information about animals’ management, phenotypic measurements, RNA-seq libraries, and initial processing can be found in Alexandre et al. (2015, 2019). In brief, RNA was extracted from samples of adrenal gland, hypothalamus, liver, skeletal muscle, and pituitary of 18 Nellore bulls including nine from each extreme of FE (evaluated by residual feed intake, Koch et al., 1963). They were part of a feeding trial containing 98 steers (16–20 months old). Of 18 bulls from eight sires and 18 dams, 14 were half-sibs from four sires. Eighty-six RNA libraries were sequenced using an Illumina HiSeq2500 equipment (2 × 100 pb). Reads were aligned to the new bovine reference genome (ARS-UCD1.2) using STAR 2.2.1 (Dobin et al., 2013). Secondary alignments, duplicated reads, and reads failing vendor quality checks were removed using Samtools (Li et al., 2009). Then, featureCounts v.3 (Liao et al., 2014) was used to generate gene read counts. EdgeR R package (Robinson et al., 2010) was used to normalize the counts by TMM (trimmed mean of M-values) and, for each tissue, only genes presenting at least 1 CPM (counts per million) in at least half of the samples were considered for the analysis. Across all five tissues, a total of 16,423 genes passed the quality check, comprising as follows: 14,158 in adrenal gland; 14,581 in hypothalamus; 12,090 in liver; 11,391 in skeletal muscle; and 13,912 in pituitary. Among them, 9,950 genes were expressed in common across all five tissues.

### Identification of Differentially Expressed Genes

Two conventional methods (*t*-test and edgeR) and three ML methods (RF, XGBoost, and RX) were used to identify subsets of potential predictor genes in individual tissues of HFE and LFE cattle. A threefold cross-validation scheme was applied within each tissue. That is, all 18 bulls (nine HFE and nine LFE) were randomly separated into three equal-size groups and each group had six cattle (three HFE and three LFE). Each group was in turn used as a testing dataset. For *t*-test and edgeR, two (12 animals) of three groups were used to derive DEG. Then the third group was used for SVM.

For RF, XGBoost, and RX, the 18 animals were randomly divided into three groups, training, validation, and testing groups (six animals each). Within each fold, six animals were left out as the testing dataset for SVM, while other two groups (six for training and six for validation) were used for RF or XGBoost or RX to select a subset of genes. The hyperparameter tuning for individual ML methods was performed with the training dataset; optimal parameters were applied to the validation dataset. Once the subsets of potential predictor genes were selected by five aforementioned methods (*t*-test, edgeR, RF, XGBoost, and RX) in each of five tissues, then these genes were evaluated for their prediction accuracy for classifying HFE and LFE animals in the testing datasets using SVM.

All analyses were performed with the R program (v3.6.1). The details of individual methods are described as follows.

#### Benchmark Tests

In this study, *t*-test and edgeR (Robinson et al., 2010) were conducted as the benchmarks to compare the performance of individual ML methods in identifying subsets of DEG for classification of HFE and LFE animals. A gene was declared DEG, if the difference in gene expression values between high (HFE) and low (LFE) groups resulted in a *P*-value < 0.05.

#### Random Forest

Random Forest is a tree-based ensemble learning method for regression or classification of multiple variables (Breiman, 2001). In general, the RF algorithm generates a multitude of individual decision trees from different bootstrap samples (i.e., subsets with replacements in both predictors and response variables), for the split (root node) in each tree. RF produces variable important measures (VIMs) for individual predictor variables. In classification problems, one way to derive the VIM for a predictor variable can be based on how much the accuracy decreases when the variable is excluded from an out-of-bag (OOB) sample of a decision tree by using a random shuffling method. The average decrease in accuracy across all trees that contain that predictor variable will be the measure of VIM value. The larger the VIM value is, the more important the variable is in the classification. All genes can be ranked with VIM values. The RF library in R software was applied for the RF analysis.

There are two crucial parameters in RF that need to be determined prior to the final RF analysis: the number of the decision trees (Ntree) in a forest and the number of predictor variables (mtry) randomly sampled as candidates for splitting at each tree node. To derive minimum hyperparameter values required, we systematically examined a range of Ntree and mtry values using training datasets of a threefold CV scheme. These included Ntree = 100,000, 200,000, 300,000, … 2,000,000 (i.e., interval = 100,000), and mtry = 1, sqrt (M), or 0.1 × M, where M is the total number of genes in each tissue. We used the error rate curve to determine the appropriate parameters for final analysis. When the error rate reaches a steady state in which its value is not affected by the increase in Ntree, then the corresponding parameter values for Ntree and mtry are determined for the RF analysis.

#### Extreme Gradient Boosting

Gradient boosting machine (GBM; Friedman, 2001) is another ensemble ML method similar to RF but with a great improvement in the prediction error. It builds a predictive model in the form of an ensemble of lots of weak learners (i.e., small subsets of predictor variables to form decision trees) in a stage-wise way. The loss function can be optimized in the function space by iteratively selecting functions that are most correlated with the negative gradient. That is, each subsequent decision tree is generated to minimize the prediction error made by the previous decision tree until no further improvements can be made.

Extreme Gradient Boosting (XGBoost; Chen et al., 2016) is very similar to GBM in principle, but it has several optimizations in the algorithm including a novel tree learning algorithm for handling sparse data, and a parallel and distributed computing which makes it more than 10 times faster and with better performance in controlling prediction errors and over-fitting problems than GBM. Similar to other decision trees methods, such as RF, XGBoost produces a VIM rank for the genes. VIM value that XGBoost produces is the “Gain.” In the current study, the Gain value of individual variable (gene) denotes the relative contribution of the gene for each tree in the model, the higher the “Gain” value is, the more important the gene is for generating a prediction.

The XGBoost library (Chen et al., 2016) in R software was applied in this study. The details of XGBoost can be seen in the guide for XGBoost (Chen and Guestrin, 2016). Two crucial tree parameters were evaluated prior to final XGBoost analysis: *eta* and *colsample*. *Eta* determines the learning rate, i.e., the rate at which a model learns patterns from decision trees. In general, the bigger the *eta* value is, the faster to learn a pattern, a higher chance to have an overfitting problem. Therefore, a smaller eta value is preferred but a trade-off between a smaller eta value and extreme high computational time needs to be considered. The colsample specifies the proportion of genes to be subsampled for a decision tree. A range of values examined in this study included: colsample = 0.1, 0.05, 0.03, and 0.01 for the three cross-validation datasets, and eta = 0.01, 0.05, 0.1, and 0.2. Again, the error rate curve was used for determining the appropriate parameters for final analysis.

#### Combination of Random Forest and XGBoost

The RX model is a two-step method of applying RF and XG. First, RF was applied to select the subset of genes with positive values of the mean decrease in accuracy, and then these selected genes from RF were further assessed by XGBoost for their associations with FE.

#### Classification of HFE and LFE Animals Using Subsets of Genes and Support Vector Machine

Support vector machine, also known as support vector networks, is a powerful supervised learning classification tool (Cortes and Vapnik, 1995). It identifies a decision boundary (hyperplane) or set of decision boundaries between two unlabeled categories in a high- or infinite-dimensional space that enables the prediction of labels from multiple features, intuitively. A perfect separation is achieved by generating the hyperplanes with the largest margin between different categories. The R library e1071 (Meyer et al., 2019) is used for the SVM analysis.

Four metrics, overall accuracy, precision, recall, and F1-score, were used for assessing the performance of SVM. They are calculated as follows:

$$\mathrm{Overall}\,\mathrm{accuracy}=\frac{\mathrm{true}\,\mathrm{positive}+\mathrm{true}\,\mathrm{negative}}{\begin{array}{c} \mathrm{true}\,\mathrm{positive}+\mathrm{false}\,\mathrm{positive}\\ +\mathrm{false}\,\mathrm{negative}+\mathrm{true}\,\mathrm{negative} \end{array}}$$

$$\text{Precision}=\frac{\mathrm{true}\,\mathrm{positive}}{\mathrm{true}\,\mathrm{positive}+\mathrm{false}\,\mathrm{positive}}$$

$$\text{Recall}=\frac{\mathrm{true}\,\mathrm{positive}}{\mathrm{true}\,\mathrm{positive}+\mathrm{false}\,\mathrm{negative}}$$

$$\mathrm{F1}\,\text{-score}=2\times \frac{\text{precision}\times \text{recall}}{\text{precision}+\text{recall}}$$

where true positive is the number of animals correctly classified by the SVM to the first category with truly observed phenotype (e.g., HFE); true negative is the number of animals correctly classified to the second category (i.e., LFE); false positive is the number of animals incorrectly assigned to the first category they do not belong (e.g., LFE animals to HFE group); and false negative is the number of animals incorrected assigned to the second category (e.g., HFE animals to LFE group). The overall accuracy is the most common metric to use and the F1-score is useful when there is an uneven distribution of different classes.

### Gene Co-expression Network Analysis

To identify significant gene to gene associations among the subsets of genes selected by the RX method with the highest classification accuracy, gene co-expression network analysis was conducted using the Partial Correlation and Information Theory (PCIT) algorithm (Reverter and Chan, 2008). The results from PCIT were visualized using Cytoscape software Version 3.7.1 (Shannon et al., 2003).

There were two types of networks constructed in the current study: within tissue and across tissues. In the within tissue approach, the expression data of the genes selected by RX in each tissue were used for the PCIT analysis. Then the correlations were calculated between the VIM values (“Gain” values) of the genes selected by RX with the values of seven major network centrality measures including betweenness, degree, closeness, clustering coefficient, neighborhood connectivity, radiality, and topological coefficient. For a detailed definition of centrality measures, see Table 1 in Junker et al. (2006). These centrality measures were computed using the Network Analyzer plugin of Cytoscape (Assenov et al., 2008).

**TABLE 1:** Hyperparameter values used in the Random Forest and XGBoost analyses.

	**RF (threefold CV)**	**XGBoost (threefold CV)**
Adrenal gland	Mtry = 0.1 × M	Colsample = 0.01
	Ntree = 2,000,000	Eta = 0.5
Hypothalamus	Mtry = sqrt (M)	Colsample = 0.01
	Ntree = 2,000,000	Eta = 0.5
Liver	Mtry = sqrt (M)	Colsample = 0.01
	Ntree = 1,000,000	Eta = 0.01
Muscle	Mtry = sqrt (M)	Colsample = 0.01
	Ntree = 1,000,000	Eta = 0.01
Pituitary	Mtry = 0.1 × M	Colsample = 0.03
	Ntree = 2,000,000	Eta = 0.01

*M, total number of genes in individual tissue; Ntree, number of trees; Mtry, number of genes for forming a decision tree; colsample, proportion of genes subsampled for a decision tree; eta, learning rate; CV, cross validation.*

In the across tissues approach, the expression data of all the genes selected by the RX and expressed in all five tissues were selected and separated into two groups (HFE and LFE) according to the animals for PCIT algorithm. The genes were considered as group-specific if the average gene expression level in one tissue was higher than those of the remaining tissues. A comparison was then performed between the gene co-expression networks of two groups, mainly focused on the group-specific connections and the differential connectivity of each gene from LFE to HFE.

### Enrichment Analysis

To further understand the biological relevance of the potential predictor genes, functional enrichment was performed using GO enrichment analysis and KEGG pathway analysis. GO enrichment analysis was performed using the program PANTHER (protein annotation through evolutionary relationship; Mi et al., 2013), and KEGG pathway analysis was performed using the program KOBAS (KO-Based Annotation System; Wu et al., 2006). A total of 16,423 genes obtained after QC were used as background, the number of genes enriched in GO and KEGG was counted, followed by Fisher’s exact test with FDR multiple test correction to assess statistical significance (adjusted *P* < 0.05).

## Results

### Hyperparameter Determination for Individual ML Methods

The final parameters used for RF and XGBoost analyses of different tissues were chosen based on a systematic evaluation of a range of values using a threefold CV and can be seen in Table 1. Different tissue datasets require different fine-tuned parameters.

### Identification of Differentially Expressed Genes Using *t*-Test, edgeR, RF, XGBoost, and RX

Using a threefold cross-validation scheme for each gene expression dataset of five tissues, we identified different numbers of DEG by *t*-test, edgeR, RF, XGBoost, and RX (Table 2). Among five methods applied, the RF produced the largest number of genes while the RX had the smallest number of genes. The number of DEG identified by *t*-test and edgeR was similar in liver, but substantially different in other four tissues (adrenal gland, hypothalamus, muscle, and pituitary gland), in which the edgeR identified more DEG than the *t*-test except for pituitary gland where the opposite was true. When comparing the selected genes by RF, XGBoost, and RX with those from *t*-test and edgeR, we found that the RF selected almost all DEG identified by *t*-test and edgeR as well as new genes (91.5 and 93.7% of the genes identified by *t*-test and edgeR were identified by RF, respectively), while the XGBoost and RX only picked up the top-ranked DEG by *t*-test and edgeR.

**TABLE 2:** Number of potential predictor genes for feed efficiency identified by *t*-test, edgeR, Random Forest (RF), Extreme Gradient Boosting (XGBoost), and the combination of Random Forest and Extreme Gradient Boosting (RX) in individual five tissues.

**Tissue**	**Total no genes**	**CV dataset**	**Method**				
			***t*-Test**	**edgeR**	**RF**	**XGBoost**	**RX**
Adrenal gland	9,581	1	115	157	2,283	72	7
		2	428	640	2,202	63	20
		3	103	252	2,369	42	6
		Total	586	941	4,993	171	33
Hypothalamus	9,810	1	46	194	1,902	88	13
		2	280	592	1,848	70	17
		3	182	290	1,812	81	8
		Total	473	908	4,041	222	33
Liver	5,005	1	50	121	905	109	17
		2	129	182	957	74	4
		3	299	294	999	66	10
		Total	421	486	2,092	227	30
Muscle	6,580	1	200	269	1,408	68	6
		2	295	322	1,676	69	9
		3	491	619	1,304	68	12
		Total	874	950	3,294	199	23
Pituitary	9,726	1	200	239	1,906	78	12
		2	684	356	1,902	58	15
		3	1,850	1,303	2,454	48	15
		Total	2,492	1,625	4,869	180	41

*CV, cross-validation; total, total number of genes identified from threefold cross-validation datasets.*

### Using SVM to Evaluate the Classification Performance of DEG Selected by Five Methods

Table 3 presents the overall accuracies and F1-scores of classification performance of different sets of DEG identified by each individual method, when applying SVM for classification. It can be seen that the classification performance varied with the genes from different tissues and different selecting methods. Regardless of the metrics used (overall accuracy or F1-score), all subsets of genes selected from five methods produced a good classification accuracy (>90% in both overall accuracy and F1-score in Table 3). When comparing the results within individual tissues, among five methods, the genes chosen by the RX showed the highest overall classification accuracy for HFE and LFE animals in hypothalamus (95.4%), liver (93.6%), muscle (96.0%), and pituitary gland (97.9%). The only exception was in the adrenal gland for which the genes selected by edgeR produced the best classification performance with 96.1% accuracy (Table 3). When comparing the average classification accuracy of five methods across all tissues (see the overall average values in Table 3), the RX outperformed the other four methods with the highest accuracy value of 95.2%. This was closely followed by *t*-test (94.8%) and edgeR (94.3%). The results for F1-score (the bottom part of Table 3) that were the weighted average of Precision and Recall values (Supplementary Table 1) showed a similar trend to that of overall accuracy values, except that the *t*-test gave the highest F1-score among all methods in the pituitary gland (F1 score, Table 3).

**TABLE 3:** Comparison of classification performances (overall accuracy and F1-score) of subsets of selected genes from different methods, when applying SVM.

**Tissue**	**Source of subset genes**					**Best**
	***t*-Test**	**edgeR**	**RF**	**XGBoost**	**RX**	
**Overall accuracy**						
Adrenal gland	0.937 (0.0719)	0.961 (0.0320)	0.902 (0.0836)	0.931 (0.0783)	0.937 (0.0768)	edgeR
Hypothalamus	0.950 (0.0278)	0.939 (0.0454)	0.945 (0.0483)	0.952 (0.0428)	0.954 (0.00641)	RX
Liver	0.932 (0.0711)	0.907 (0.0605)	0.933 (0.0387)	0.918 (0.0679)	0.936 (0.0534)	RX
Muscle	0.945 (0.0312)	0.937 (0.0446)	0.942 (0.0245)	0.925 (0.0518)	0.960 (0.0414)	RX
Pituitary	0.978 (0.00789)	0.973 (0.0204)	0.978 (0.00581)	0.972 (0.00554)	0.979 (0.00701)	RX
Overall average	0.9484	0.9434	0.9400	0.9396	0.9532	RX
**F1-score**						
Adrenal gland	0.949 (0.0551)	0.956 (0.0396)	0.915 (0.0714)	0.937 (0.0702)	0.949 (0.0598)	edgeR
Hypothalamus	0.948 (0.0289)	0.945 (0.0469)	0.947 (0.0440)	0.948 (0.0482)	0.951 (0.00871)	RX
Liver	0.930 (0.0732)	0.886 (0.0845)	0.927 (0.0457)	0.897 (0.0931)	0.932 (0.0575)	RX
Muscle	0.948 (0.0367)	0.940 (0.0442)	0.945 (0.0298)	0.924 (0.0577)	0.957 (0.0442)	RX
Pituitary	0.988 (0.0195)	0.973 (0.0205)	0.978 (0.0161)	0.958 (0.0197)	0.977 (0.00518)	*t*-Test
Overall average	0.9526	0.9400	0.9424	0.9328	0.9532	RX

*Values in brackets are standard deviations.*

**FIGURE 1:**
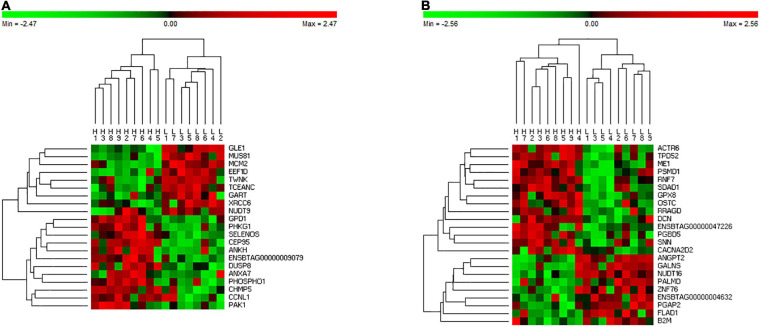
Heatmap of cluster analysis using DGE identified by the RX in muscle **(A)** and pituitary gland **(B)**. H refers to HFE bulls and L refers to LFE bulls.

Given that RX identified the smallest subsets of potential predictor genes across all tissues with the highest classification accuracy for nine HFE and nine LFE animals, a further investigation in gene expression pattern was carried out for the 160 genes selected by the RX from different tissues (i.e., 33, 33, 30, 23, and 41 genes from adrenal gland, hypothalamus, liver, muscle, and pituitary, respectively). Figure 1 illustrates the cluster analysis heatmaps of 18 animals using the 23 genes from muscle (Figure 1A) and 41 genes from pituitary gland (Figure 1B), respectively. It can be seen the distinguishable pattern between the HFE and LFE animals in both tissues even with the very small sets of genes (Figure 1), especially in muscle (Figure 1A). The heatmaps for other tissues can be seen in Supplementary Figure 1.

### Gene Co-expression Networks

#### Gene Co-expression Network Within Individual Tissues

**FIGURE 2:**
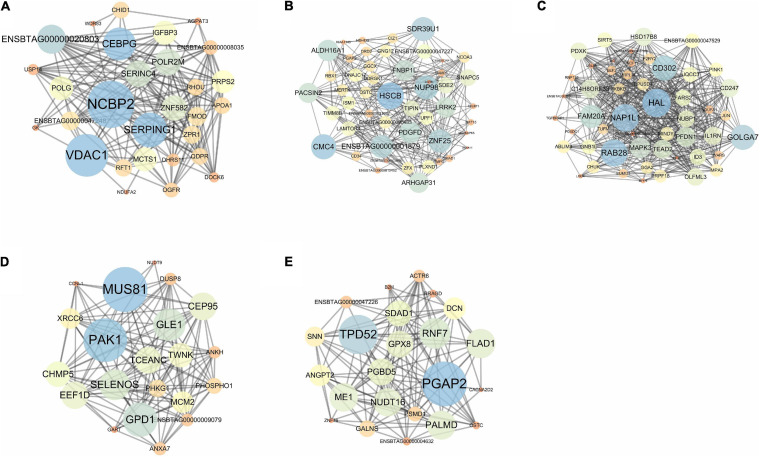
Co-expression network of predictor genes identified by the RX in adrenal gland **(A)**, hypothalamus **(B)**, liver **(C)**, muscle **(D)**, and pituitary **(E)**. Deep color (blue) and bigger circles indicate the genes with stronger control power (higher betweenness value) over the network.

The five individual co-expression networks (Figure 2) were composed of 29, 49, 51, 21, and 23 potential predictor genes and 179, 285, 489, 135, and 133 connections in adrenal gland (Figure 2A), hypothalamus (Figure 2B), liver (Figure 2C), muscle (Figure 2D), and pituitary (Figure 2E), respectively.

Pearson correlation coefficient (PCC) analysis was conducted between the “Gain” values from RX and seven major centralities in the gene co-expression network; the results are shown in Table 4. Across all five tissues, betweenness (the degree to which nodes stand between each other) had the highest average correlation coefficient (0.21) and all above 0.10, followed by degree (number of the connections of each node) (0.18).

**TABLE 4:** Correlations between ML “Gain” values from RX analysis and network centrality parameters.

	**Betweenness**	**Closeness**	**Clustering**	**Degree**	**Neighborhood**	**Radiality**	**Topological**
Adrenal gland	0.10	0.30	0.11	0.30	0.27	0.29	0.03
Hypothalamus	0.23	0.09	−0.14	0.09	−0.04	0.08	−0.13
Liver	0.23	0.25	−0.08	0.29	0.06	0.23	−0.03
Muscle	0.29	−0.01	−0.10	0.00	−0.13	0.02	−0.19
Pituitary	0.21	0.25	−0.11	0.24	−0.09	0.23	−0.13
AVERAGE	0.21	0.17	−0.06	0.18	0.01	0.17	−0.09

#### Gene Co-expression Networks Across Five Tissues for HFE and LFE Groups

**FIGURE 3:**
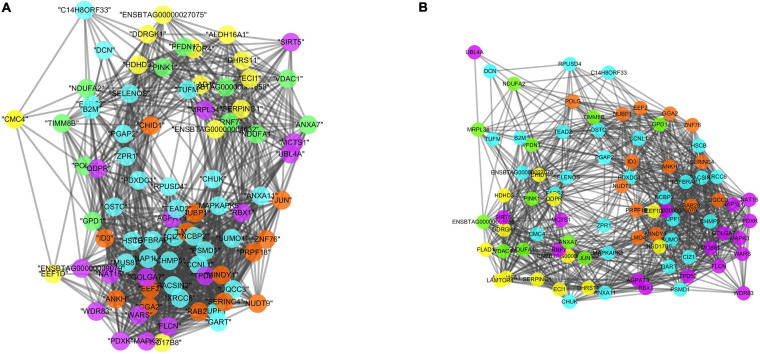
Co-expression networks in LFE **(A)** and HFE **(B)**, colors are relative to the tissue of maximum expression: yellow represents liver, green represents muscle, orange represents pituitary, purple represents hypothalamus, and blue represents adrenal gland. The results are based on the genes selected by the RX.

When considering the five tissues altogether, two gene co-expression networks were constructed, one for LFE animals (Figure 3A) and one for HFE animals (Figure 3B), based on the genes present in different tissues with the highest expression values among five tissues. Of the 84 genes, 31 were from adrenal gland, 16 from hypothalamus, 12 from liver, 11 from muscle, and 14 from pituitary.

The LFE and HFE specific networks were composed of 1,056 and 1,129 connections, respectively. When comparing the connections within tissues, there were 45.31, 40.32, and 15% more connections created for hypothalamus, adrenal gland, and pituitary of the HFE network than those in LFE network. Conversely, there were 95.3 and 35.31% less connections created in HFE than connections lost in LFE in muscle and liver.

**FIGURE 4:**
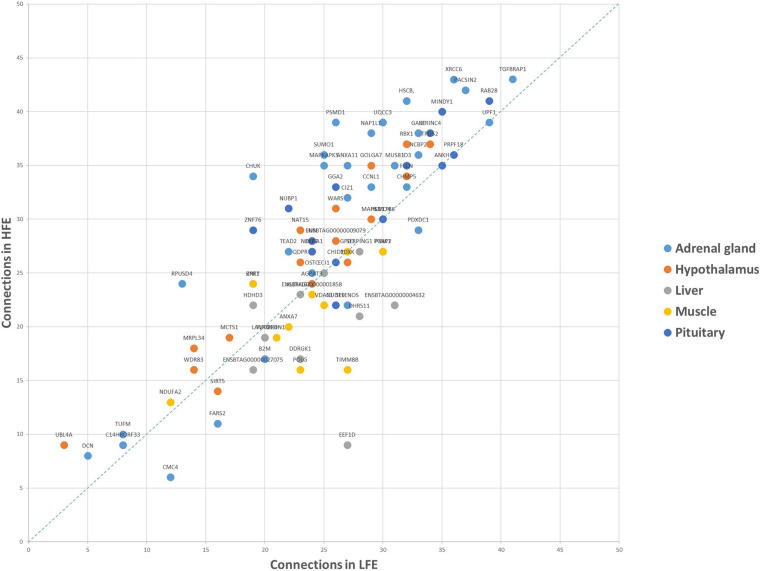
Connections of each shared gene in LFE and HFE. The results are based on the genes selected by the RX.

Regarding the connections of each gene (Figure 4), the top five most connected regulators were *TGFBRAP1, RAB28, PACSIN2, XRCC6*, and *UPF1*, varying from 84 to 78 connections in two groups, the top five regulators with the biggest change in the number of connections were *EEF1D, CHUK, PSMD1, RPUSD4*, and *SUMO1* varying from 18 to 11.

### Enrichment Analysis

Table 5 presents the results from the GO enrichment analyses of the genes selected by RX in all five tissues, using the *Bos taurus* reference from the PANTHER program. A total of 21 GO terms were significantly enriched and the top five enriched GO terms included metabolic process (GO:0008152), cellular metabolic process (GO:0044237), organic substance metabolic process (GO:0071704), primary metabolic process (GO:0044238), and nitrogen compound metabolic process (GO:0006807).

**TABLE 5:** GO enrichment analysis based on genes selected by RX.

**GO biological process complete**	**Number of genes**	***P*-value**
Metabolic process (GO:0008152)	86	1.47E-07
Cellular metabolic process (GO:0044237)	75	2.89E-07
Organic substance metabolic process (GO:0071704)	73	2.51E-05
Primary metabolic process (GO:0044238)	69	3.94E-05
Nitrogen compound metabolic process (GO:0006807)	64	6.74E-05
Biosynthetic process (GO:0009058)	35	1.39E-06
Organic cyclic compound metabolic process (GO:1901360)	35	1.97E-05
Cellular biosynthetic process (GO:0044249)	34	6.62E-07
Organic substance biosynthetic process (GO:1901576)	34	1.67E-06
Cellular aromatic compound metabolic process (GO:0006725)	32	6.66E-05
Phosphate-containing compound metabolic process (GO:0006796)	27	6.85E-05
Response to oxygen-containing compound (GO:1901700)	22	2.71E-06
Macromolecule biosynthetic process (GO:0009059)	22	6.20E-05
Cellular response to oxygen-containing compound (GO:1901701)	19	1.08E-06
Positive regulation of intracellular signal transduction (GO:1902533)	17	2.14E-05
Organic cyclic compound biosynthetic process (GO:1901362)	17	7.40E-05
Regulation of anatomical structure morphogenesis (GO:0022603)	17	8.20E-05
Response to oxidative stress (GO:0006979)	10	3.17E-05
Cellular response to oxidative stress (GO:0034599)	8	3.11E-05
Cellular response to cadmium ion (GO:0071276)	5	1.32E-06
Response to cadmium ion (GO:0046686)	5	2.96E-06

Table 6 presents the top 20 enriched pathways from the KEGG analyses of genes selected by RX in all five tissues, using the *Bos taurus* reference from the KOBAS program. The top five enriched pathways were metabolic pathways (hsa01100), MAPK signaling pathway (hsa04010), Ras signaling pathway (hsa04014), T cell receptor signaling pathway (hsa04660), and Parkinson’s disease (hsa05012).

**TABLE 6:** KEGG enrichment analysis based on genes selected by RX.

**Pathways**	**Number of genes**	***P*-value**
Metabolic pathways (KEGG:01100)	16	2.29E-05
MAPK signaling pathway (KEGG:04010)	9	7.79E-07
Ras signaling pathway (KEGG:04014)	7	3.23E-05
T cell receptor signaling pathway (KEGG:04660)	6	4.17E-06
Parkinson’s disease (KEGG:05012)	6	2.17E-05
mTOR signaling pathway (KEGG:04150)	6	3.36E-05
Chemokine signaling pathway (KEGG:04062)	6	9.50E-05
PI3K-Akt signaling pathway (KEGG:04151)	6	2.11E-03
Pathways in cancer (KEGG:05200)	6	4.32E-03
Renal cell carcinoma (KEGG:05211)	5	8.01E-06
TGF-beta signaling pathway (KEGG:04350)	5	2.26E-05
Chagas disease (KEGG:05142)	5	5.98E-05
Osteoclast differentiation (KEGG:04380)	5	1.76E-04
Influenza A(KEGG:05164)	5	6.32E-04
Epithelial cell signaling in Helicobacter pylori infection (KEGG:05120)	4	1.62E-04
B cell receptor signaling pathway (KEGG:04662)	4	2.10E-04
Prostate cancer (KEGG:05215)	4	4.32E-04
Toll-like receptor signaling pathway (KEGG:04620)	4	8.12E-04
TNF signaling pathway (KEGG:04668)	4	9.28E-04
Apoptosis (KEGG:04210)	4	2.19E-03

## Discussion

Feed efficiency is a complex phenotype, regulated by several biological processes, such as feed intake, digestion, metabolism, physical activity, and thermoregulation (Herd and Arthur, 2009). Therefore, accurately predicting FE and related traits from molecular datasets is not straightforward. So far, several studies have explored the feasibility of identifying molecular predictors for FE using different ML algorithms [e.g., Clemmons et al., 2019 (Beef cattle), Messad et al., 2019 (pigs), and Piles et al., 2019 (pigs)]. However, none of these attempted to compare the prediction performance of combining two ML methods together. In this study, using SVM, we evaluated classification performance for FE based on the subsets of selected genes by five different methods including RF, XGBoost, RX, edgeR, and *t*-test. The reason why RF, XGBoost, and SVM were applied in this study not ENET is that RF, XGBoost, and SVM are the most commonly used decision-trees-based ML methods for regression or classification; they are easier to apply than ENET. In addition, for proof of concept of combining ML methods together, we chose to apply RX (combining RF and XGBoost). SVM was used as the judge because the similar results were observed with SVM to that of RF when initially testing individual methods.

Across five methods in all tissues, higher average accuracy and F1-score values obtained in pituitary indicate that the genes identified in pituitary produced clear expression pattern difference between HFE and LFE animals. To date, many studies of FE focused on liver tissue; our results suggest that pituitary could be an important tissue to investigate as well. Although the subsets of genes chosen by all methods produced good overall classification accuracy (>90%), the number of genes varied significantly. The RX method produced the highest value of prediction accuracy yet with the smallest subsets of genes (117) that were biologically relevant to FE. This is in stark contrast with the other methods in this study that require large number of genes to achieve similar accuracy values. This has great implication in future when considering the efficiency and the cost of determining the number of genes required for classifying animals of different FE. In addition, the reasons why the RX performed the best can be explained as follows:

A prediction error from a supervised learning algorithm consists of two parts: a bias and a variance (James et al., 2013). A bias is “the persistent or systematic error that the learning algorithm is expected to make when trained on training sets of size *m*” (Dietterich and Kong, 1995). It is the difference between predicted values using training sets and the expected true value. A variance refers to the variation of predicted values for all individuals of a given dataset (Olaru and Wehenkel, 2004). In other term, it indicates the amount by which the fitted model would change when different training sets were applied. Prediction errors of different boosting and bagging decision tree methods have different characteristics. In general, boosting methods (such as XGBoost), based on ensemble of weak learners (i.e., lots of decision trees with small numbers of predictor features), produce the results with a high bias but a low variance. In contrast, bagging trees (such as Random Forest), produce an outcome with a low bias and high variance (Podgorelec et al., 2015; Liam et al., 2018). Given the individual method’s shortcomings, by analyzing the gene expression data first by RF to choose the features with positive VIM values (>0) and then applying XGBoost to select final subsets of predictor genes for classification using SVM, we were able to take full advantages of what each method can offer to minimize the prediction error. This explains why our combined method, the RX, had the best performance among all methods. Furthermore, our results confirmed the findings by Xiong et al. (2019) that combining ML models could provide a better accurate assessment model than individual ML model alone, particularly in the context of complex animal production traits.

Of the five methods tested, surprisingly Random Forest had the worst performance in terms of overall accuracy of classifying HFE from LFE animals when comparing with the results from *t*-test and edgeR, despite the fact that RF identified the largest number of potential predictor genes. Fernandez-Delgado et al. (2014) evaluated the accuracy of 179 ML methods including Bayesian approaches, neural networks, SVM, boosting, bagging, and others in 121 datasets at both large and small scales for bio and non-bio problems. For multi-class datasets, RF was found to outperform all other methods and achieved on average 94.1% of the accuracy. For two-class datasets, their study showed that SVM was the best (95% of the accuracy) followed closely by the RF (94.3%). One explanation for our results is that using the simple criteria of VIM > 0, the majority of the subset of genes chosen by RF were not significant DEG, by including these genes in the classification prediction model presented a larger prediction error than those significant DEG genes chosen by *t*-test and edgeR using *P* < 0.05.

For validating the biological reliability of RX results, we conducted co-expression network analyses using the DE genes selected by RX and calculated the PCCs between the “Gain” values from RX and seven major centrality measures of the gene co-expression network in each of the five tissues. Among those, betweenness centrality, a measure that shows the status of a gene in connecting two or more groups of genes, presented the highest PCCs. This suggests that the genes with higher “Gain” also have more control over the network, because more information passes through them (Godini and Fallahi, 2018). These bottle-neck nodes in the network reflect an important regulatory role for the phenotype under study, providing a good connection between the genes identified by RX for FE. This is the first study to combine ML and gene co-expression network analysis to confirm the interactivity existing among these genes and their relevance within the network related to their prediction ranking based on ML.

When comparing co-expression network differences between LFE and HFE groups, although the number of connections in both groups was similar, there were more connections in HFE than in LFE. At tissue level, the number of connections between genes with maximum expression in skeletal muscle represented the biggest change between HFE and LFE networks, with more connections being created in the HFE network. Our results imply that there may be more FE-related pathways activated in HFE, particularly at the level of skeleton muscle. Regarding the connections of each gene, the topmost connected regulator was *TGFBRAP1* (transforming growth factor beta receptor associated protein 1), which encodes a protein that binds to transforming growth factor-beta (TGF-beta) receptors and plays a key role in TGF-beta signaling pathway. *TGFB1* has been previously found as a key regulator of FE using this same dataset and a multi-tissue co-expression network comprised of 1,335 relevant genes for this trait (Alexandre et al., 2019) and using a completely different phenotype-metabolome-genome integrated dataset (Widmann et al., 2015). The regulator with the biggest change in the number of connections between HFE and LFE was *EEF1D* (eukaryotic translation elongation factor 1 delta), a crucial activator of Akt-mTOR signaling pathway (Cheng et al., 2018).

To further understand the function of the genes identified by RX (Supplementary Table 2), we performed GO and KEGG enrichment analyses. The most enriched terms were metabolic process and metabolic pathways in GO and KEGG, respectively. These complex biological processes are related with FE, among which, metabolic pathways are known to play an important role in controlling of FE. Previous studies have revealed that variation in metabolic pathways leads to variation of FE (Abo-Ismail et al., 2013; Saatchi et al., 2014; Abasht et al., 2019). Furthermore, pathways and processes related to metabolic pathways were also found enriched in previous FE studies in cattle (Santana et al., 2014) and pig (Onteru et al., 2013). Other pathways worth citing include mTOR signaling pathway, PI3K-Akt signaling pathway, and TGF-beta signaling pathway which have been reported to be highly related to FE in other studies (Hill and Azain, 2009; Sartin, 2013). These results further demonstrate the additional value of using RX in generating biological insights.

It is cautionary to mention the limitations of our study, regarding the number of ML methods tested and the sample size of the RNA-seq dataset. The small number of animals (18) and close relationship between them (half-sibs) in the training, validation, and testing datasets could explain why high classification accuracy values achieved for all the methods. The results could be different if the training, validation, and testing groups are not closely related, especially in the case where a population is small and there is large individual variation. To minimize the influence of small population size and large individual variation on prediction accuracy of ML methods, one of the methods is to apply Leave-One-Out Cross-Validation scheme (Sammut and Webb, 2011). The utility of combining different ML methods needs to be further validated considering other traits with different heritability values, livestock species, and populations.

## Conclusion

In summary, using expression data of 16,432 genes in five tissues from 18 Nellore bulls, we demonstrate that: (1) combining Random Forest and XGBoost (RX), a two-step ML method, has great potential in identifying small subsets of biologically important genes for accurately classifying FE animals and (2) a correlation exists among the genes identified by RX in their relevance to the networks and their prediction ranking by RX. The findings from this study are not only relevant to FE, but also have great potential implications to the study of other important complex traits in cattle as well as in other livestock species.

## Data Availability Statement

Publicly available datasets were analyzed in this study. These data can be found here: European Nucleotide Archive under the study ID PRJEB27337 (https://www.ebi.ac.uk/ena/data/view/PRJEB27337).

## Ethics Statement

Ethical review and approval was not required for the animal study because the dataset used for the study is available in the public domain.

## Author Contributions

YL and AR: conceptualization and supervision. WC, PA, and GR: formal analysis. HF: data curation. WC, PA, AR, and YL: writing, review, and editing. WS: funding acquisition. All authors contributed to the article and approved the submitted version.

## Conflict of Interest

The authors declare that the research was conducted in the absence of any commercial or financial relationships that could be construed as a potential conflict of interest.

## Footnotes

1 https://www.ebi.ac.uk/ena/data/view/PRJEB27337

## Supplementary Material

Supplementary Figure 1 Heatmap of predictor genes identified in adrenal gland, hypothalamus, and liver.

Supplementary Table 1 Comparison of classification performances (precision and recall) of subsets of differentially expressed genes selected from different methods, when applying SVM.

Supplementary Table 2 List of potential predictor genes identified in different tissues by RX.
