# Supplementary figures and images for: Identification of Predictor Genes for Feed Efficiency in Beef Cattle by Applying Machine Learning Methods to Multi-Tissue Transcriptome Data
Source: Front Genet. 2021 Feb 16;12:619857. doi: 10.3389/fgene.2021.619857 (PMC7921797; doi:10.3389/fgene.2021.619857)

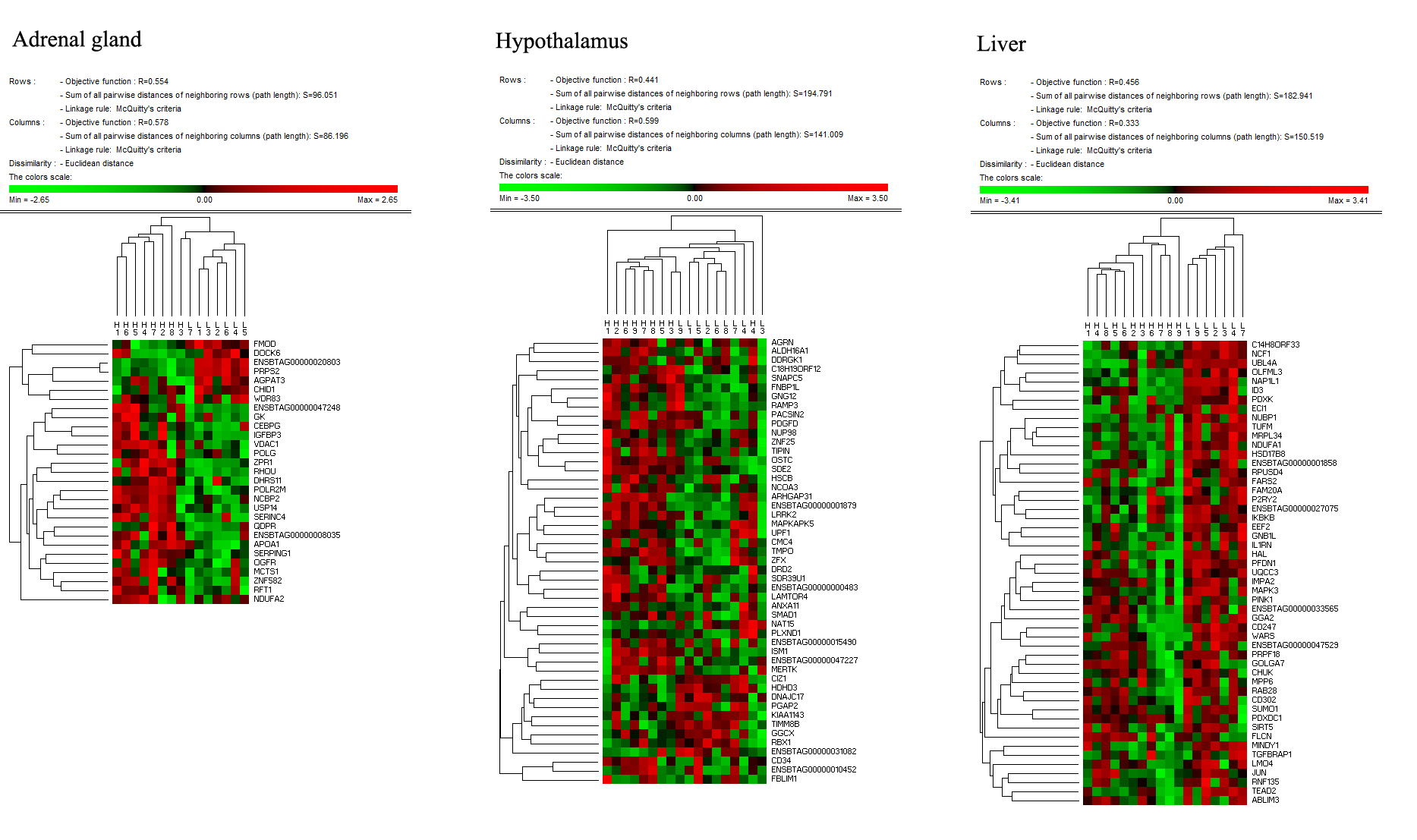

Supplement: Supplementary Figure 1 — Heatmap of predictor genes identified in adrenal gland, hypothalamus, and liver. [file Data_Sheet_1.ZIP › supplmentary/Supp_ Figure 1.jpg]
